# Combining Kangaroo Care and Live-Performed Music Therapy: Effects on Physiological Stability and Neurological Functioning in Extremely and Very Preterm Infants

**DOI:** 10.3390/ijerph18126580

**Published:** 2021-06-18

**Authors:** Loïs C. Span, Nienke H. van Dokkum, Anne-Greet Ravensbergen, Arend F. Bos, Artur C. Jaschke

**Affiliations:** 1 Division of Neonatology, Department of Pediatrics, Beatrix Children’s Hospital, University Medical Center Groningen, University of Groningen, 9713 GZ Groningen, The Netherlands; n.h.van.dokkum@umcg.nl (N.H.v.D.); a.g.ravensbergen@umcg.nl (A.-G.R.); a.f.bos@umcg.nl (A.F.B.); a.c.jaschke@umcg.nl (A.C.J.); 2 Department of Music Therapy, ArtEZ University of the Arts, 7523 WB Enschede, The Netherlands

**Keywords:** live-performed music therapy, kangaroo care, general movements, general movement optimality score

## Abstract

Interventions such as kangaroo care (KC) and live-performed music therapy (LPMT), are increasingly used to facilitate stress reduction in neonates. This study aims to investigate the effect of combining the two on physiological responses and neurological functioning in very preterm infants. Infants received six sessions of LPMT. KC was added to one LPMT session. Physiological responses included heart rate, respiratory rate and oxygen saturation. We videotaped infants for 30 min before and after two sessions to assess general movements (GMs). We included 17 infants, gestational age median 26.0 weeks (IQR 25.6–30.6 weeks), of whom six were males. Combined interventions showed a decrease in heart rate from mean 164 bpm before to 157 bpm during therapy, *p* = 0.001. Oxygen saturation levels increased during combination therapy from median 91.4% to 94.5%, *p* = 0.044. We found no effects of LPMT or combined interventions on GMs. Infants with a postnatal age (PNA) <7 days generally seem to display less optimal GMs after therapy compared with infants with a PNA >7 days. In conclusion, combining interventions is equally beneficial for physiological stability and neurological functioning as LPMT alone. Future studies should focus on the effects of this combination on parent-infant bonding.

## 1. Introduction

Prematurity is a global concern, with 10.6% of all live births in 2014 ending before 37 weeks of gestation. This comprises over 14.9 million infants globally, with preterm birth rates increasing in most countries [1,2]. In Europe, the percentage of very preterm births (<32 weeks’ gestation) ranges from 0.7 to 1.4% of all live births [3]. Very preterm infants are at higher risk of adverse outcomes, with 10–15% infant mortality and 5–10% cerebral palsy [3,4]. Survival rates of extremely and very preterm infants are also increasing due to technological advances and the combined efforts of obstetricians and neonatologists worldwide [5]. Still, complications of preterm birth are the leading cause of infant mortality and morbidity in almost all countries [1]. Shortened gestation predisposes preterm infants to a wide array of complications. Preterm infants are at higher risk for neurodevelopmental impairments [6,7] as well as behavioral [8] and attentional problems [9]. The tiniest and most fragile infants are at highest risk of these disabilities [10], with a 40% prevalence being reported for infants delivered at 24–32 weeks gestational age (GA) [11]. The focus for antenatal, perinatal and neonatal interventions has therefore diverted from minimalization of death rates to the optimization of preterm infant care, to reduce long-term morbidity, especially the prevention of brain injury and abnormal brain development [9].

Several interventions are provided to these infants during their stay in the Neonatal Intensive Care Unit (NICU). One of the most commonly implemented interventions is kangaroo care (KC), where skin-to-skin contact between infants and parents is considered as a key part of care. Numerous studies have reported on the benefits of KC related to physiological stability, that is thermoregulation, stable cardiorespiratory functions and blood glucose levels, as well as improved sleep-wake cycles and higher rates of breastfeeding [12,13,14]. KC is also an effective therapy to relieve procedural pain and improve neurodevelopment [12,13,14,15]. Therefore, KC has become a popularized strategy worldwide for promoting parent-infant attachment and for modifying NICU distress.

Over recent years, evidence is rapidly accumulating that music-based interventions may also positively impact preterm infants’ physiological stability and behavioral states [16,17,18,19,20,21]. Studies report that music aids in the stabilization of heart rate, blood pressure and respiration [18,20], as well as the improvement of oral feeding tolerance [17,22,23]. Recent research suggests that the best practice of music therapy is live-performed music therapy (LPMT) as it is an individual approach adjusted to the infants’ physiological and behavioral state with encouragement of parental participation [19,23,24]. LPMT can be defined as “a clinical and evidence-based health intervention in which music is used within a therapeutic setting to address patients’ physical, emotional, cognitive, and social needs” [19]. LPMT is a feasible intervention that is tolerated well and does not cause overstimulation in extremely and very preterm infants [25,26].

The individual KC and LPMT interventions have therefore proven their value in neonatal care [25,27]. Nonetheless, only a few studies exist that investigate the combined effects of musical interventions and KC [24,28,29,30,31]. These studies generally reported stable or slightly beneficial changes in physiological parameters during and after therapy. However, these studies mostly used music-based interventions i.e., recorded music, live-harp music and maternal singing, rather than LPMT, and include mainly older infants. Consequently, the combined effects of LPMT and KC in extremely and very preterm infants have been studied insufficiently. Therefore, the aim of this study was to investigate the added value of combining KC and LPMT compared with LMPT alone on physiological stability and neurological functioning in extremely and very preterm infants.

## 2. Materials and Methods

### 2.1. Setting and Population

For this prospective, single-center, within-subject, comparative crossover study, we included infants born between September 2020 and April 2021. All clinically stable infants, born with a gestational age of less than 32 weeks, combined with a birth weight of <2000 g, who were cared for in the level III-IV NICU of the University Medical Center Groningen, The Netherlands, were eligible for inclusion. Exclusion criteria were: (a) medical instability (e.g., continuing bradycardias, apneas, saturation drops or acute illness) (b) significant congenital anomalies, (c) intraventricular hemorrhages grade 3 or 4, (d) heavy sedation and (e) surgical intervention until five days after. Nurses and neonatologists were consulted for risk assessment of individual cases. Parents needed to have a sufficient understanding of the Dutch or English language to understand the purpose of this research, give informed consent, and schedule appointments for the study interventions. Parents were approached during the first week after birth to participate in the study and were provided with written and oral information about the study. Infants were included in the study after both parents provided written informed consent. This study was conducted within an ongoing pilot trial concerning the feasibility and effects of LPMT for extremely and very preterm infants. Because of its pilot nature, we did not perform a sample size calculation. This study’s ethical approval was obtained by the institutional ethical review board of the University Medical Center Groningen (METc 2019/093) and was registered online (ISRCTN94562698).

### 2.2. Procedure

After enrolment, a certified music therapist, who specialized in NICU music therapy, provided detailed information on the LPMT as provided at our neonatal ward. Infants in the music therapy trial received six sessions of LPMT consisting of roughly 15 min of actual music. Two of these sessions, provided on different days, were used for the current study, one consisting of LPMT only and one where LPMT was provided during a KC session.

#### 2.2.1. Music Therapy

A full explanation of the music therapy trial has been described previously [25]. In summary, LPMT was based on the Rhythm, Breath and Lullaby method, as developed by Loewy [23]. While observing the infants’ vital signs, the music therapist played improvised melodies using two musical interventions. The type of instrument was carefully selected for each session and depended on the gestational age and developmental progress. The first instrument we used was the Remo Lullaby Ocean Disc, a round drum with metal balls inside that simulates womb sounds. The second instrument we used was live singing accompanied by guitar arpeggios. The Ocean Disc was used in the first three sessions. After the third session, guitar arpeggios and voice were introduced. Parents were encouraged to provide their song of kin, i.e., a meaningful song that the music therapist rewrote to resemble a lullaby. If a song of kin was not provided, Twinke Twinkle Little Star was the lullaby of choice.

#### 2.2.2. Kangaroo Care

KC is standard practice in Dutch neonatal wards. The preterm infant is clad in a diaper and is held in a vertical upright position against the parent’s bare chest while covered with clothing or a blanket. Parents are motivated to provide KC as soon as the infant is medically stable. Skin-to-skin contact was provided for a minimum of 60 min a day. One of the LPMT sessions was scheduled during such a 60 min period. After the 15 min of LPMT, parents continued KC for a minimum of 30 min.

### 2.3. Measures

#### 2.3.1. Physiological Stability

We determined physiological stability using heart rate, respiratory rate and peripheral oxygen saturation. For all three, we calculated means in the hour before therapy, during therapy and in the hour after therapy. Data on physiological parameters were extracted from bedside monitors, in which they were stored in five second intervals.

#### 2.3.2. General Movement Assessment

We determined neurological functioning using the General Movement Assessment (GMA), including the detailed General Movement Optimality Score (GMOS). The Prechtl GMA is one of the most reliable indicators for early neuromotor deficits and reflects impairments of brain regions involved in neurodevelopment [32,33,34,35]. The GMA describes the quality and quantity of the spontaneous motor repertoire. We recorded infants before and after both interventions for a minimum of 30 min. Infants were recorded in supine or lateral recumbent position, preferably without clothing and distracting objects. From the recordings, we first assessed the quality of General Movements (GMs) based on visual gestalt perception, differentiating normal GMs from abnormal GMs, in the categories poor repertoire, cramped synchronized and chaotic movements. Next, the GMOS was applied to all recordings, where the subcategories, i.e., neck and trunk, upper extremities and lower extremities, were scored. The GMOS ranges between 5 and 42 points, with higher scores indicating better neurological performance [34]. All scoring was performed by two trained and licensed GM assessors (N.H.v.D. and A.F.B.), who were blinded for patient characteristics and the type of intervention.

### 2.4. Statistical Analysis

Analyses were performed using SPSS version 27.0 (IBM Statistics, Armonk, NY, USA). First, we reported baseline characteristics of infants using descriptive statistics. Baseline characteristics included gender, birth weight, gestational age, postnatal age during intervention, and respiratory support. Next, we compared differences in heart rate, respiratory rate and peripheral oxygen saturation before, during and after therapy sessions with one another, using Wilcoxon’s signed-rank test. Finally, we assessed differences in GMOS before and after therapy sessions and tested these using Wilcoxon’s signed rank test. We also tested the differences in delta GMOS of LPMT alone compared with LPMT and KC together, using Wilcoxon’s signed rank test. Results with *p* < 0.05 were considered statistically significant.

## 3. Results

### 3.1. Patient Characteristics

Overall, 50 infants were eligible to participate in this study. Of these, two could not be asked for participation because of logistical reasons, three infants deceased and ten were transferred to other hospitals before participation could be asked. Of the remaining 35 eligible infants, the parents of six declined participation for this study. Consequently, this study included 29 infants, of which 17 completed both interventions. They had a median gestational age of 26.0 weeks (Interquartile Range (IQR) 25.6–30.6 weeks) and median birth weight 900 g (IQR 766–1380 g). We present the baseline characteristics in Table 1. The twelve infants who were transferred to other care facilities before the interventions could be completed did not statistically differ regarding GA (*p* = 0.093), birth weight (*p* = 0.141) and male-female ratio (*p* = 0.096).

### 3.2. Physiological Parameters

#### 3.2.1. Heart Rate

**Figure 1:**
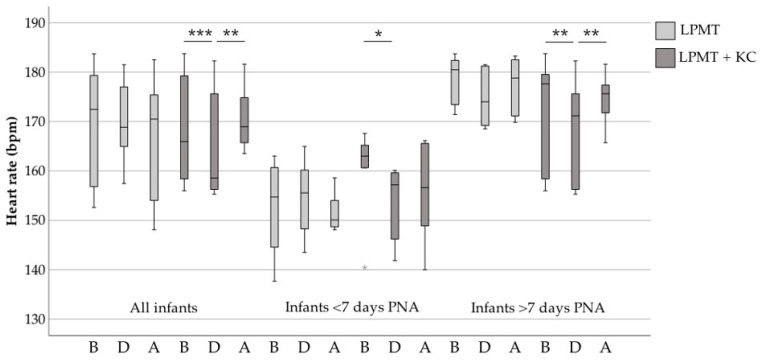
Heart rate before, during and after LPMT compared with combined interventions. The boxes represent the interquartile ranges and the whiskers represent the range of all infants. Grey stars indicate the outliers of this study. PNA, Postnatal age; LPMT, live-performed music therapy; KC, kangaroo care; Bpm, beats per minute; B, before therapy; D, during therapy; A, after therapy; *, *p* < 0.05; **, *p* ≤ 0.01; ***, *p* ≤ 0.001.

In Figure 1 we present the differences between the heart rate of all included infants, infants <7 days postnatal age (PNA) and infants >7 days PNA. Combined interventions showed a significant decrease in heart rate during therapy (median 164 bpm before to median 157 bpm during therapy, *p* = 0.001). A significant increase of heart rate was observed after combined therapy (median 157 bpm during to median 166 bpm after, *p* = 0.01). Heart rate before, during and after LPMT alone remained stable (*p* = 0.25 to 0.91 for all three comparisons). In infants with a PNA <7 days, heart rate seemed to rise during LMPT alone, whereas combined with KC, it seemed to drop. 

#### 3.2.2. Respiratory Rate

**Figure 2:**
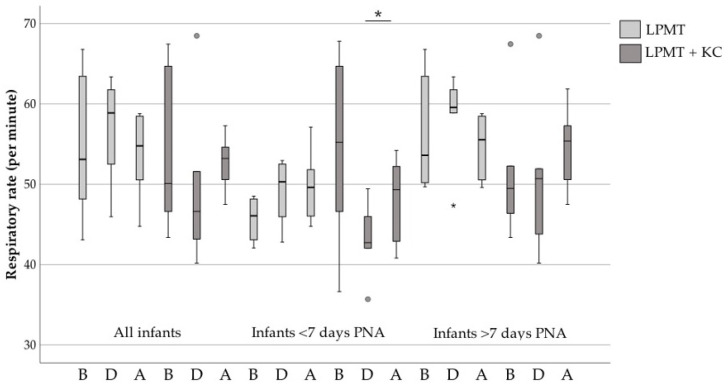
Respiratory rate before, during and after LPMT compared with combined interventions. The boxes represent the interquartile ranges and the whiskers represent the range of all infants. Grey points and stars indicate the outliers of this study. PNA, postnatal age; LPMT, live-performed music therapy; KC, kangaroo care; B, before therapy; D, during therapy; A, after therapy; *, *p* < 0.05.

In Figure 2 we present the results on respiratory rate in infants of all postnatal ages, <7 days PNA and >7 days PNA. There were neither significant changes in the respiratory rate in the LPMT group, nor were there any significant changes in the combined therapy group of all infants (*p* = 0.16 to *p* = 0.89). Respiratory rate increased significantly after combined interventions in the <7 days PNA group compared with during therapy (*p* = 0.046). Although not statistically significantly different, respiratory rate seemed to drop during combined interventions, whereas it seemed to rise during LPMT.

#### 3.2.3. Peripheral Oxygen Saturation

**Figure 3:**
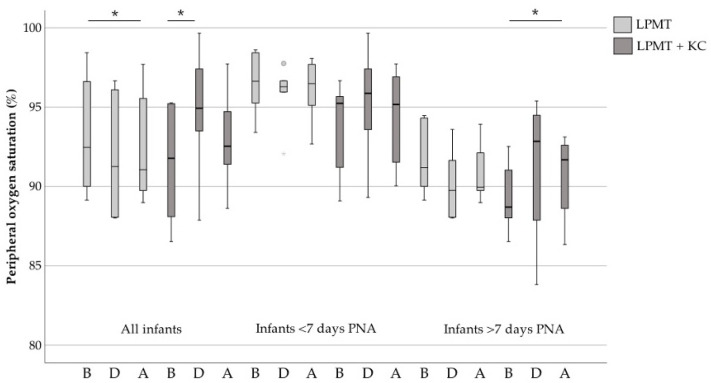
Oxygen saturation before, during and after LPMT compared with combined interventions. The boxes represent the interquartile ranges and the whiskers represent the range of all ages. Grey points and stars indicate the outliers of this study. LPMT: live-performed music therapy KC: kangaroo care; B, before therapy; D, during therapy; A, after therapy; *, *p* < 0.05.

In Figure 3, we present the responses of all included infants compared with infants that received therapy before and after 7 days PNA. Oxygen saturation decreased statistically significantly after LPMT (median 94.0% before to median 92.7% after, *p* = 0.011). Regarding combined interventions, we observed an increase of oxygen saturation during therapy (median 91.4% before to median 94.5% during, *p* = 0.044). This oxygen saturation increase during LPMT combined with KC was significant in infants older than 7 days PNA (*p* = 0.033). Although not statistically significant, oxygen saturation levels of preterm infants seemed to slightly elevate during combined therapy and slightly drop during LPMT alone in both PNA groups.

### 3.3. Neurological Functioning

**Figure 4:**
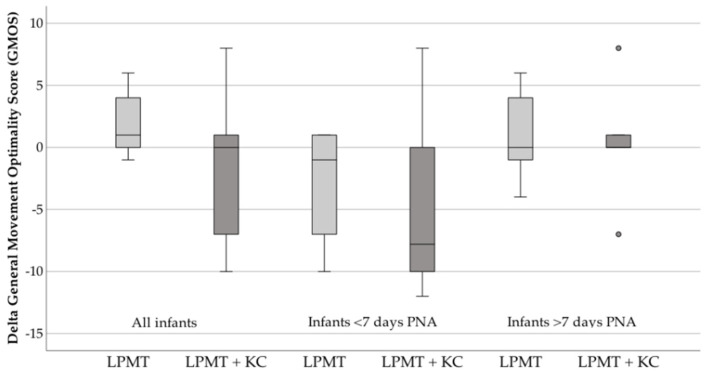
Delta General Movement Optimality Score (GMOS) for all infants, infants <7 days PNA and infants >7 days PNA. Grey points indicate the outliers of this study. GMOS, General movement optimality score; LPMT, live-performed music therapy; KC, kangaroo care; PNA, postnatal age.

We analyzed a total of 68 recordings, 17 before and 17 after LPMT and compared these recordings with 17 before, and 17 after LPMT combined with KC. We present GMs patterns of individual recordings in Table 2. One infant scored hypokinetic on two of the recordings, so we excluded this infant for GMA. Most infants’ GMs were scored ‘poor repertoire’. There was no statistically significant difference between the pre–post-therapy in GMOS in infants provided with LPMT (31.3 vs. 29.6, *p* = 0.20) or combined interventions (28.2 vs. 27.6, *p* = 0.86). Comparing the delta GMOS’ for both interventions also revealed no significant differences (Figure 4). 

Because of the vast differences in GMOS scores, we decided post-hoc to investigate the patterns of GMs in detail. We noticed that infants with a PNA <7 days had more irregular GMOS’ than infants >7 days PNA, although this could not be supported statistically (Figure 4).

## 4. Discussion

This study investigated the effects of LPMT during KC compared with LPMT alone on physiological stability and neurological functioning in extremely and very preterm infants. We demonstrated that combining LPMT and KC was beneficial for physiological stability, illustrated by a decrease in heart rate of roughly 7 bpm during therapy, as well as an improved oxygen saturation. Our results did not show effects of either LPMT or combined interventions on GMs. Preterm infants who received therapy with a PNA of <7 days generally displayed less optimal GMOS’ after therapy than infants with a PNA of >7 days. The key points of our study are summarized in Table 3.

We found a decreased heart rate during combined interventions, which was not evident during LPMT alone. This finding is in line with those of Teckenberg-Jansson and colleagues, who concluded that live music therapy during KC has a positive impact on physiological parameters, particularly heart rate, during therapy in infants between 24 and 36 weeks of GA [24]. A recent meta-analysis examining the effects of music therapy alone also did not detect heart rate changes after therapy [20]. These outcomes might imply that the beneficial effects of combination therapy are largely the effect of KC alone. KC provides a multisensory stimulation, including emotional, tactile, visual and auditory stimuli and is proven to be beneficial for autonomic functioning, improved mother-infant interaction and better sleep wake cycles [36]. Parent-infant synchrony offers a unique co-regulatory framework for stabilizing physiological parameters and improving neurodevelopmental outcomes that might cause decreases in heart rate during combination therapy [37,38,39].

We did not identify effects of either intervention on respiratory rate. This finding is similar to other studies reporting continued stability of respiratory rate during combination therapy and LPMT alone [18,22,30,31,40]. In our study, respiratory rate seemed to slightly increase during LPMT alone and slightly decrease during LPMT combined with KC. This trend was visible for both PNA groups. None of these analyses regarding respiratory rate reached statistical significance, except for one and all respiratory rates were within normal ranges for preterm infants. Ultimately, baseline outcomes of all participating infants did not show any differences during or after both therapies, indicating that respiratory rate remained unchanged.

Regarding oxygen saturation, we found a slight decrease after LPMT alone. Oxygen saturation slightly increased during combination therapy. Ettenberger and colleagues, as well as Yusuf and colleagues also found an increase in oxygen saturation during combination therapy [28,29]. The decrease after LPMT that we found is in contrast with several recent meta-analyses investigating the physiological effects of music therapy alone, which reported improvement or stabilization of oxygen saturation [18,20,41,42]. Of note, our finding of only a 1.3% lower peripheral oxygen saturation is, although statistically significant, clinically irrelevant. Slight variations in peripheral oxygen saturation in preterm infants are common, and 1.3% falls withing the range of measurement error considered to be around 3–4% [43]. Findings may also be biased, because we did not specifically record the ventilatory oxygen changes made by nurses during and after therapy. Even so, the incongruity between both interventions suggests that combination therapy has a more favorable effect on oxygen saturation than LPMT alone.

Overall, our findings indicated improved physiological stability when comparing combined interventions with LPTM alone. These effects were only short-term, as after combined interventions physiological parameters returned to initial values. Regardless of the variation in the methodological approach, other studies investigating the effects of combination therapy generally also reported stable or slightly beneficial changes in vital parameters during and after combination therapy [24,28,29,30,31,40]. Previous studies have reported that both KC and music therapy are well accepted, cost-effective, accessible, and safe methods that improve physiological parameters and neurodevelopmental outcomes, and reduce infant stress [13,18,20]. The possible effect of KC is expected to be greatest in low-resource countries, because other options for care of preterm infants remain limited. Despite the high impact and apparent feasibility of KC and LPMT, few preterm infants in low-resource countries currently have access to KC, let alone LPMT [14]. Our findings therefore contribute to the growing body of evidence on the effectiveness of KC and LPMT, but also raise questions about worldwide feasibility. Future research could focus on the feasibility of these interventions in low recourse settings.

One of our most important findings was that there was no difference in GMOS before and after interventions, which indicates no immediate effect on neurological functioning for either form of therapy. We were unable to confirm the findings of Bos and colleagues, who reported a vast increase in GMOS from before to after therapy [44]. Infants in our study were, however, slightly younger than infants in their study, which may explain this difference. The similar persistent stability of GMs in both therapies suggests that LPMT during KC is also feasible in extremely and very preterm infants admitted to the NICU. Potentially, there could be clinical advantages to the synergic effects of combination therapy that were not investigated or established in this study. Over the years, numerous studies have demonstrated beneficial outcomes for individual treatments of KC and music therapy regarding neurodevelopmental outcomes, parent-infant bonding, and stress reduction in both infants and parents [15,18,20,38,45,46,47,48]. Future studies should aim to further clarify the effects of LPMT alone and in combination with KC on neurological functioning in the short-term and long-term.

Strikingly, we found that infants reacted better to LPMT therapy after 7 days PNA when compared with receiving therapy before 7 days PNA. The mean delta GMOS of infants before 7 days PNA was primarily negative for both interventions, whereas for infants after 7 days PNA the delta was predominantly positive, indicating better neurological outcomes after 7 days PNA. The differences found between PNA groups are intriguing and warrant further analysis. Previous studies found that the GMA is a good indicator for brain functioning and short-term neurodevelopmental outcomes in very preterm infants [32,33,49,50,51]. However, it has also been reported that detailed GMOS scoring may be inconsistent and influenced by many unknown factors during the first week after birth [51,52,53,54]. It may be that potential positive effects of LPMT during the first week on neurological functioning are concealed by this varying GMOS. Still, we also cannot rule out that LPMT is not apt for the tiny infant during the first days after birth, due to the transition from fetal to neonatal period, even if morbidities are not so severe. Then, perhaps we should postpone LPMT until 7 days after birth, at least until future studies have elucidated this further.

### Strengths and Limitations

The main strength of this research is the structured approach in which parents and preterm infants were provided with LPMT and KC. LPMT sessions were provided by the same certified music therapist and performed on fixed times and dates. The most significant limitation of this study is the small sample size. Small intervention groups complicate the study’s reliability because it may lead to higher variability. However, the results of our findings were relatively consistent with previous literature and we consider them valid. Another limitation of our study is the lack of a control group of KC alone. A control group could have provided a clearer overview of the defined effects of KC, LPMT and combination therapy. Future research should distinguish the isolated effects of both individual therapies and compare them both to a combined intervention. Finally, there was some variety in timeframe between filming GMs and therapy. We tried to systematically film infants, but not every infant could be recorded in the same timespan before or after therapy because of logistical reasons, e.g., medical procedures, standard care and availability of the music therapist. We only measured infants at a specific moment and did not follow them over a more extended period. These short-term observations make this research more prone to selection bias. Research investigating the long-term effects of combination therapy on neurodevelopmental outcomes is highly warranted.

## 5. Conclusions

In conclusion, this study supports the clinical use of LPMT alone and LPMT during KC in extremely and very preterm infants admitted to the NICU. Combining LPMT with KC may be slightly more beneficial for physiological stability and equally effective for neurological functioning. We advise that LPMT should be postponed until 7 days after birth. Future large-scale studies are highly needed to further clarify the effects of LPMT and combination therapies and should focus on long-term neurodevelopment and parent-infant bonding.

## Figures and Tables

**Table 1:** Baseline patient characteristics for infants that completed both interventions.

Characteristic	*N* = 17
Gestational age (weeks)	26.0 (25.6–30.6)
Birth weight (grams)	900 (766–1380)
Sex	
Male	6 (35)
Female	11 (65)
Kangaroo care	
Mother	11 (65)
Father	6 (35)
Respiratory support during LPMT	
Mechanical ventilation	1 (6)
CPAP	11 (65)
High flow/low flow/no support	5 (29)
Respiratory support during LPMT + KC	
Mechanical ventilation	5 (29)
CPAP	7 (41)
High flow/low flow/no support	5 (29)
PNA during first therapy session (days)	17.0 (5.5–26.0)
Time between filming and start intervention (min)	55 (12–115)
Time between end intervention and filming (min)	25 (15–60)

LPMT: Live-performed music therapy CPAP: Continuous Positive Airway Pressure. KC: Kangaroo Care. PNA: postnatal age. Min: minutes. Data are presented as median (interquartile range) or *n* (%) where appropriate. Percentages do not always add up to 100 because of rounding.

**Table 2:** The GMOS and quality of general movements per individual infant before and after LPMT alone and combined interventions.

Infant	GMOS LPMT			GMOS LPMT during KC		
Number	Before	After	Delta	Before	After	Delta
1	PR (34)	PR (33)	↓	N (38)	N (39)	↑
2	N (32)	PR (32)	=	PR (25)	PR (25)	=
3	N (36)	N (40)	↑	N (33)	PR (26)	↓
4	PR (27)	PR (33)	↑	PR (22)	PR (30)	↑
5 *	PR (26)	PR (25)	↓	H (No score)	H (No score)	NA
6	PR (31)	PR (32)	↑	N (36)	PR (26)	↓
7	PR (33)	PR (23)	↓	PR (33)	PR (25)	↓
8	PR (32)	PR (28)	↓	PR (25)	PR (25)	=
9	PR (32)	PR (25)	↓	PR (19)	PR (27)	↑
10	PR (31)	PR (32)	↑	PR (34)	PR (22)	↓
11	PR (31)	PR (30)	↓	PR (25)	PR (25)	=
12	PR (23)	N (35)	↑	PR (21)	PR (28)	↑
13	N (38)	PR (27)	↓	PR (28)	PR (29)	↑
14	PR (30)	PR (25)	↓	N (33)	N (35)	↑
15	PR (32)	PR (30)	↓	PR (31)	PR (32)	↑
16	PR (32)	PR (25)	↓	PR (28)	PR (21)	↓
17	PR (26)	PR (23)	↓	PR (22)	PR (26)	↑

GMOS: general movement optimality score (ranges between 5 and 42), LPMT: live-performed music therapy, KC: kangaroo care, N, normal; PR, poor repertoire; H, hypokinetic; IQR, interquartile range; NA, not applicable; ↑, increase; =, no change; ↓, decrease; * Was not used in statistical analysis, because hypokinetic GMs could not be scored.

**Table 3:** Key points of this study.

Combining LPMT and KC was beneficial for physiological stability. No effect on GMOS was found for either LPMT or combined interventions. Preterm infants who received therapy with a PNA of <7 days generally displayed less optimal GMOS’ after therapy than infants with a PNA of >7 days LPMT during KC is also feasible in extremely and very preterm infants admitted to the NICU. We advise to postpone LPMT until 7 days after birth. Future studies should aim to further clarify the effects of LPMT alone and in combination with KC on neurological functioning.

LPMT, Live-performed music therapy; KC, Kangaroo Care; GMOS, General Movement Optimality Score; PNA, Postnatal age; NICU, Neonatal Intensive Care Unit.

## Data Availability

The data presented in this study are available on request from the corresponding author. The data are not publicly available due to their containing information that could compromise the privacy of research participants.
